# COVID-19: first long-term care facility outbreak in the Netherlands following cross-border introduction from Germany, March 2020

**DOI:** 10.1186/s12879-021-06093-9

**Published:** 2021-05-04

**Authors:** Mitch van Hensbergen, Casper D. J. den Heijer, Petra Wolffs, Volker Hackert, Henriëtte L. G. ter Waarbeek, Bas B. Oude Munnink, Reina S. Sikkema, Edou R. Heddema, Christian J. P. A. Hoebe

**Affiliations:** 1 Department of Sexual Health, Infectious Diseases, and Environmental Health, South Limburg Public Health Service, PO-Box 33, 6400 AA Heerlen, The Netherlands; 2 Department of Social Medicine, Care and Public Health Research Institute (CAPHRI), Faculty of Health, Medicine and Life Sciences, Maastricht University, PO-Box 616, 6200 MD Maastricht, The Netherlands; 3 Department of Medical Microbiology, Care and Public Health Research Institute (CAPHRI), Faculty of Health, Medicine and Life Sciences, Maastricht University Medical Centre (MUMC+), PO-Box 5800, 6202 AZ Maastricht, The Netherlands; 4 Department of Viroscience, Erasmus MC - University Medical Center Rotterdam, Wytemaweg 80, 3015 CN Rotterdam, The Netherlands; 5 Department of Medical Microbiology, Zuyderland Medical Center Heerlen/Sittard-Geleen, 6162 BG Geleen, The Netherlands

**Keywords:** Coronavirus, SARS-CoV-2, Whole genome sequencing, Cross-sectional, Observational, Nursing home, Infection control

## Abstract

**Background:**

The Dutch province of Limburg borders the German district of Heinsberg, which had a large cluster of COVID-19 cases linked to local carnival activities before any cases were reported in the Netherlands. However, Heinsberg was not included as an area reporting local or community transmission per the national case definition at the time. In early March, two residents from a long-term care facility (LTCF) in Sittard, a Dutch town located in close vicinity to the district of Heinsberg, tested positive for COVID-19. In this study we aimed to determine whether cross-border introduction of the virus took place by analysing the LTCF outbreak in Sittard, both epidemiologically and microbiologically.

**Methods:**

Surveys and semi-structured oral interviews were conducted with all present LTCF residents by health care workers during regular points of care for information on new or unusual signs and symptoms of disease. Both throat and nasopharyngeal swabs were taken from residents suspect of COVID-19, based on regional criteria, for the detection of SARS-CoV-2 by Real-time Polymerase Chain Reaction. Additionally, whole genome sequencing was performed using a SARS-CoV-2 specific amplicon-based Nanopore sequencing approach. Moreover, twelve random residents were sampled for possible asymptomatic infections.

**Results:**

Out of 99 residents, 46 got tested for COVID-19. Out of the 46 tested residents, nineteen (41%) tested positive for COVID-19, including 3 asymptomatic residents. CT-values for asymptomatic residents seemed higher compared to symptomatic residents. Eleven samples were sequenced, along with three random samples from COVID-19 patients hospitalized in the regional hospital at the time of the LTCF outbreak. All samples were linked to COVID-19 cases from the cross-border region of Heinsberg, Germany.

**Conclusions:**

Sequencing combined with epidemiological data was able to virtually prove cross-border transmission at the start of the Dutch COVID-19 epidemic. Our results highlight the need for cross-border collaboration and adjustment of national policy to emerging region-specific needs along borders in order to establish coordinated implementation of infection control measures to limit the spread of COVID-19.

**Supplementary Information:**

The online version contains supplementary material available at 10.1186/s12879-021-06093-9.

## Background

Rapid global spread of the novel coronavirus SARS-CoV-2 has led to an increasing number of cases of coronavirus disease 2019 (COVID-19) in the Netherlands. Since the first case of COVID-19 in the Netherlands was reported on February 27, 2020 [1], COVID-19 has spread within the general population, with initial major clusters in the provinces of Brabant and Limburg, and later countrywide [2–4]. In the beginning of the outbreak, most cases were identified in returning travellers, mainly from northern Italy. Containment efforts included testing of suspected cases, contact tracing, isolation of cases and quarantine of contacts, as well as medical monitoring of exposed individuals. Suspected cases in that stage of the pandemic were defined by symptoms of fever, with cough or dyspnoea, together with an epidemiological link, i.e. contact with a laboratory-confirmed case, or prior visit to a COVID-19 endemic area [3, 5]. On February 26th, in addition to the previous case definition, diagnostic testing for COVID-19 for all hospitalized patients with unexplained pneumonia was introduced. These tests were performed in dedicated and certified labs in the Netherlands.

The Dutch province of Limburg borders the German district of Heinsberg in the federal state of North Rhine-Westphalia which had a large cluster of COVID-19 cases linked to local carnival activities, even before the first cases were reported in the Netherlands [6]. An immunocompromised man had attended several densely-packed carnival celebrations in the town of Gangelt (in the Heinsberg region) on February 15th 2020 [6]. He developed progressive respiratory symptoms shortly after, and was eventually hospitalized on February 24th in deteriorating condition, testing positive for COVID-19 on February 25th [7]. The outbreak in Heinsberg was likely fuelled by large crowds of carnival revellers (superspreading event) being exposed to this immunocompromised, supposedly super-spreading patient [8].

On March 1st 2020, the first long-term care facility (LTCF) resident in Sittard, a Dutch town located in close vicinity to the district of Heinsberg, started experiencing moderate respiratory symptoms, but did not meet the national test criteria for COVID-19. Two LTCF residents were admitted on the 5th and the 6th of March to two different wards of the regional hospital with a suspected respiratory infection, but no (initial) pneumonia. Even though they did not fit the national COVID suspect criteria, they were tested for COVID-19 and tested positive. The Public Health Service South Limburg received first reports of two COVID-19 positively tested residents on March 7th 2020.

Introduction of the virus could have occurred following the carnival activities in the surrounding area by LTCF visitors or health care workers (HCWs). This assumption is fuelled by the fact that the border region of Sittard (The Netherlands) and Heinsberg (Germany) is typified for its cross-border travel. Therefore, these densely-packed carnival activities were most likely visited by HCWs, as well as citizens from both sides of the border.

Our study group has conducted several studies regarding infectious disease control along the Dutch-German border region [9, 10], highlighting the need for specific cross-border measures and intensified cross-border collaboration. Although cross-border measures are crucial to COVID-19 response [11, 12], to our knowledge, only few studies have been published about COVID-19 transmission and challenges in border regions. One of these studies assessed cross-border transmission of the SARS-CoV-2 virus in the border regions of Venezuela with Colombia and Brazil, and reported similar genome sequences of SARS-CoV-2 despite measures in place to limit mobility, including a lockdown [13]. A recently published British study attributed pre-crisis commute to work between districts as a major cause for SARS-CoV-2 spread during the first wave of the pandemic [14], which is a scenario with similar conditions compared to the present study.

In this manuscript, we have determined whether cross-border transmission from the Heinsberg region took place by analysing the first COVID-19 LTCF outbreak in the Netherlands, located in Sittard, both epidemiologically and microbiologically. In particular, we compared SARS-CoV-2 sequences of samples found in this LTCF, as well as three samples from the regional hospital, with sequences from Heinsberg. In doing so, we want to address the challenges of a cross-border region and highlight the importance of specific, regional guidelines and needs in limiting the introduction of pathogens from across the border.

## Methods

We performed a cross-sectional epidemiological and laboratory investigation of an ongoing COVID-19 outbreak that occurred in a LTCF in South Limburg, the Netherlands, in early March 2020. Surveys were conducted with all present residents during regular points of care. On the day of survey, HCWs from the LTCF performed semi-structured oral interviews of all 99 present LTCF residents, divided over five different wards, to collect information on age, sex, new or unusual signs and symptoms of disease, complemented with comorbidity information from their patient records and taking their temperature (rectally) in the morning and the evening during regular moments of care. An increase in temperature from 37.5 to 38 degrees Celsius was categorized as subfebrile temperature, whereas 38.0 C and above was classified as fever. Optimal collection of signs and symptoms was hampered by the fact that part of the residents had some stage of impaired cognition.

These data were shared with the Public Health Service South Limburg. No HCWs were tested, as HCWs who experienced any symptoms of disease were instructed to stay at home. Twelve random samples of possible asymptomatic residents were taken from residents from all five wards to avoid possible selection bias. Any missing data was coded as missing and was not imputed. All descriptive analyses were done using IBM SPSS Statistics version 26 (IBM, Armonk, NY, USA).

### Case definition

Both international and national case definition for suspect cases, at the time, included a sudden onset of either cough, fever, shortness of breath with no other aetiology that fully explains the clinical presentation. Additionally, a suspected case must have met an epidemiological criterium of a history of travel or residence in a country/area reporting local or community transmission, or have had to be in close contact with a confirmed, or probable COVID-19 case [5]. Within Dutch hospitals, patients were also suspected of COVID-19 when they were diagnosed with pneumonia with unknown cause irrespective of an epidemiological link. At the start of March 2020, Heinsberg was not included as one of these areas in the national case definition. Therefore, in Limburg, we included travel to or from Gangelt (in the Heinsberg region) after intensive local contact (not being only buying fuel or food) as an adjusted regional case definition. Because of the cluster of two COVID-19 positively tested cases, we used a more sensitive case definition for COVID-19 suspected cases for the residents of the LTCF involved, i.e. any respiratory symptoms or fever, including subfebrility.

Suspect residents of the LTCF (*n* = 10), according to this adjusted case definition for COVID-19, were sampled on March 8th, which resulted in six confirmed COVID-19 cases on March 9th. Subsequent screening of contacts (*n* = 14) in the same ward as the index patient and on other wards revealed additional COVID-19 cases. Additionally, a random sample of twelve asymptomatic residents were tested on March 11th.

### Test analysis

Both throat and nasopharyngeal swabs were taken from residents suspect of COVID-19 for the detection of SARS-CoV-2 by Real-time Polymerase Chain Reaction (RT-PCR) [15]. In addition to samples of residents who tested positive for COVID-19, samples from COVID-19 positive cases who were admitted at the Zuyderland Medical Center (i.e. the regional hospital) in the same time period, but unrelated to the LTCF, were also analysed to evaluate the relatedness of these strains with those from the LTCF and the German region of Heinsberg. Regarding the RT-PCR, RNA was extracted from the samples with the use of automated total nucleic acid extraction using the MP96 (Roche Diagnostics, Rotkreuz, Switzerland) per the manufacturer’s instructions. In-house RT-PCR was performed using a Quantstudio 5 (Applied Biosystems, MA, USA) based on the dual-target PCR published by Corman et al. [15] targeting the E-gene and RNA-dependent RNA polymerase (RdRp). For PCR, a 20 μl PCR reaction was used, including 5 μl Taqpath 1-step RT mastermix (Applied Biosystems), 100–800 nM of primers and probes and 10 μl extracted RNA. Before extraction, all samples were spiked with murine cytomegalovirus (CMV) RNA, which was used as an extraction and amplification control. The Cycle Threshold (CT) values of the identified strains were also determined.

### Whole genome sequencing

Whole genome sequencing was performed using a SARS-CoV-2 specific amplicon-based Nanopore sequencing approach [3]. Sequence reads were demultiplex using Porechop (https://github.com/rrwick/Porechop) after which a reference-based alignment was performed using minimap2 [16]. The consensus genome was determined using custom scripts as described by Oude Munnink et al. [3].

All available full-length SARS-CoV-2 genomes were retrieved from GISAID (supplementary) on the 17th of March 2020 and aligned with the Dutch SARS-CoV-2 sequences from this study using MUSCLE [16]. Unfortunately, we could not incorporate a table to credit all contributing and submitting labs due to the huge number of people who have contributed. Sequences with > 10% “Ns” were excluded. The alignment was manually checked for discrepancies after which IQ-TREE [17] was used to perform a maximum likelihood phylogenetic analysis under the GTR + F + I + G4 model as best predicted model using the ultrafast bootstrap option with 1000 replicates. Nucleic acid sequence data has been shared with the Global Initiative on Sharing All Influenza Data (GISAID) database.

## Results

Table 1 shows the characteristics of the 99 interviewed residents, as well as the test coverage per ward and the results.

**Table 1:** Descriptive statistics of resident characteristics by ward level (1–5) from the LTCF (*N* = 99)

	Ward 1		Ward 2		Ward 3		Ward 4		Ward 5		Total	
	n/N^a^	%	n/N^a^	%	n/N^a^	%	n/N^a^	%	n/N^a^	%	n/N^a^	%
Sex												
Female	13/18	72	12/19	63	15/18	83	14/17	82	18/27	66	72/99	73
Age in years (mean, standard deviation)	83, 8.8		84, 7.5		84, 8.5		90, 4.1		87, 4.7^b^		86, 7.1	
Tested^c^												
Yes	5/18	28	4/19	21	14/18	78	6/17	35	17/27	63	46/99	47
Positive^d^	1/5	20	3/4	75	7/14	50	1/6	17	7/17	41	19/46	41
Asymptomatic	0/1	0	2/3	67	1/7	14	0/1	0	0/7	0	3/19	16
Case fatality rate	0/1	0	1/3	33	1/7	14	0/1	0	3/7	43	5/19	26

^a^ Unless stated otherwise

^b^ Age was missing for 2 residents on ward 5

^c^ Including the random sample of twelve asymptomatic residents

^d^ Based on the number of residents who were tested

The ages of the residents ranged from 64 to 97 years. Out of the 99 residents, 46 (47%) were tested for COVID-19 of whom nineteen (41%) tested positive. More tests were done on wards three and five, because COVID-19 was first detected here. Out of the twelve randomly tested asymptomatic residents, three (25%) tested positive for COVID-19.

Up until 8 weeks after the last infection was determined, five residents have died. All five deceased residents tested positive for COVID-19.

### Symptoms

Out of the nineteen residents who tested positive for COVID-19, sixteen (84%) reported any signs and symptoms of disease. Out of these sixteen symptomatic residents, we were unable to fully evaluate the symptoms of three (19%) residents, as they were hospitalized and passed away before the interviews took place. The most common signs and symptoms in the remaining thirteen positive residents were fever (54%), subfebrile temperature (47%), and cough (39%). Other, less frequently reported symptoms, were fatigue (15%), malaise (15%), vomiting (8%), loss of appetite (8%), nausea (8%), and dizziness (8%). An overview of these reported symptoms within symptomatic COVID-19 positively tested residents is shown in Table 2.

**Table 2:**
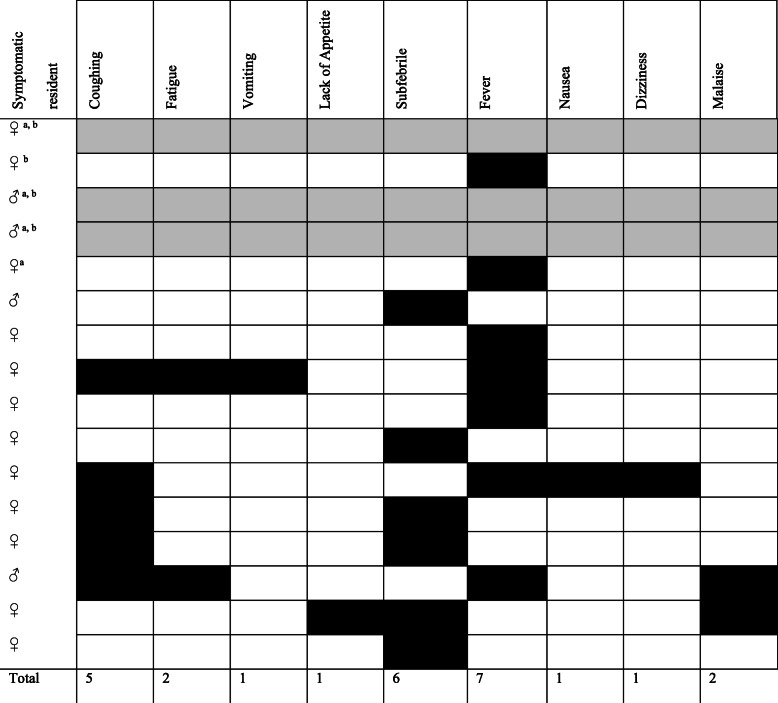
An overview of reported symptoms from symptomatic COVID-19 positive residents, March 2020 (*n* = 16)

^a^ Resident was hospitalized

^b^ Resident has died

Present symptoms are depicted with a black cell. Fever is defined as a temperature of 38.0 degrees Celsius or higher, whereas subfebrile temperature is defined as a temperature below 38.0 degrees Celsius. Three residents were hospitalized before an interview could be conducted, and are depicted with grey cells. Out of the five residents who tested positive for COVID-19 and passed away, four reported any symptoms of disease

Additionally, another eighteen residents reported an increase in temperature, with seven reporting fever and eleven reporting subfebrility, but tested negative for COVID-19.

### CT values

**Fig. 1:**
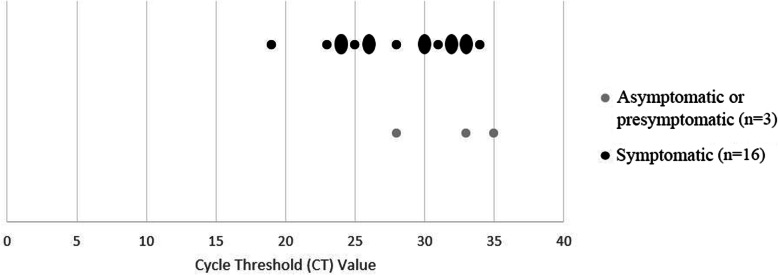
CT values of COVID-19 positive tested LTCF residents, March 2020 (*n* = 19). Oval dots represent two residents with the same CT *value*

The CT values of positively tested symptomatic residents (*n* = 16) and asymptomatic residents (*n* = 3) are shown in Fig. 1. CT values ranged from 19 up to 35; median CT values were 33 and 29 for asymptomatic and symptomatic residents respectively. Symptomatic residents appeared to have a slightly higher load (lower CT value) compared to asymptomatic residents.

### Genome sequencing

Complete genome sequencing of the SARS-CoV-2 virus from eleven residents from the LTCF in South Limburg was performed. In addition, three random samples from the regional hospital were sequenced. All accession numbers for the sequences are shown in Table 3.

**Table 3:** Sequencing names and accession numbers, March 2020 (*n* = 14)

Name	Accession number	Location	Collection date	Data source
Limburg_7	EPI_ISL_415464	Europe / Netherlands / Limburg	March 2020	GISAID
Limburg_13	EPI_ISL_461001	Europe / Netherlands / Limburg	March 2020	GISAID
Limburg_14	EPI_ISL_461002	Europe / Netherlands / Limburg	March 2020	GISAID
Limburg_15	EPI_ISL_461003	Europe / Netherlands / Limburg	March 2020	GISAID
Limburg_66	EPI_ISL_461051	Europe / Netherlands / Limburg	March 2020	GISAID
Limburg_67	EPI_ISL_461052	Europe / Netherlands / Limburg	March 2020	GISAID
Limburg_68	EPI_ISL_461053	Europe / Netherlands / Limburg	March 2020	GISAID
Limburg_69	EPI_ISL_461054	Europe / Netherlands / Limburg	March 2020	GISAID
Limburg_70	EPI_ISL_461055	Europe / Netherlands / Limburg	March 2020	GISAID
Limburg_71	EPI_ISL_461056	Europe / Netherlands / Limburg	March 2020	GISAID
Limburg_72	EPI_ISL_461057	Europe / Netherlands / Limburg	March 2020	GISAID
Limburg_73	EPI_ISL_461058	Europe / Netherlands / Limburg	March 2020	GISAID
Limburg_74	EPI_ISL_461059	Europe / Netherlands / Limburg	March 2020	GISAID
Limburg_75	EPI_ISL_461060	Europe / Netherlands / Limburg	March 2020	GISAID

**Fig. 2:**
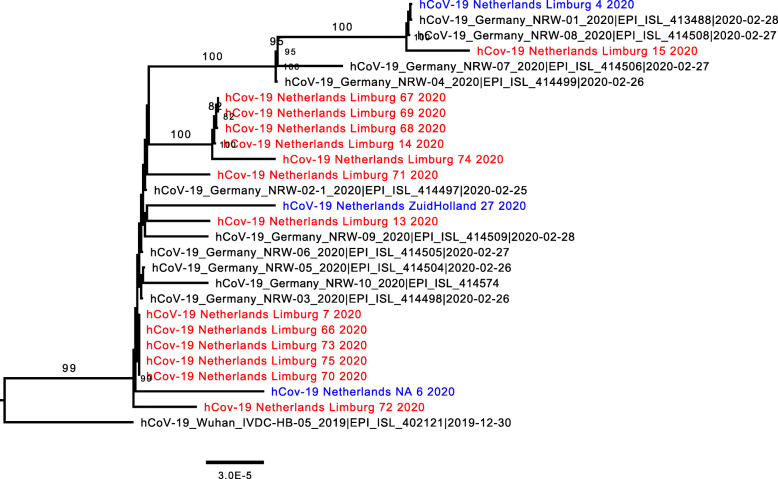
Zoom in of the full phylogenetic tree on the Dutch LTCF residents from the LTCF in Sittard, the Netherlands, March 2020 (*n* = 14). The scale bar represents the number of substitutions per site. Sequences from the Netherlands are depicted in blue, and specific sequences from this study in red. Samples 13, 14, and 15 depicted in red were samples acquired from hospitalized patients in the regional hospital around the same time of the LTCF outbreak. The remaining red samples were acquired from eleven COVID-19 positive LTCF residents

Figure 2 shows that the sequences from the LTCF residents in Sittard are part of the same cluster, pointing towards potential transmission within the nursing home. The sequences also cluster with sequences found in the hospitalized patients as well as in Heinsberg, implicating regional cross-border spread of this strain. The full phylogenetic tree can be found in the supplementary (Additional file 1).

### Outbreak control measures

As of March 8th 2020, additional infection control measures were implemented by the LTCF. Wards three and five, the wards on which the first residents tested positive for COVID-19, were isolated, and visitors were banned from entering these wards. Admission of new residents was temporarily halted. All suspect residents (residents with respiratory complaints with and without fever) and confirmed COVID-19 residents, as well as residents with (at least) subfebrility, were put into isolation. All other residents were closely monitored by taking their temperature twice during regular moments of care, as well as checking for new or unusual signs and symptoms of disease, including coughing, subfebrility, and shortness of breath. Additionally, all planned group activities on all wards were cancelled, and residents were served meals in their own rooms.

At first, the number of visitors was limited to two per day for the other wards. However, when residents tested positive for COVID-19 on these remaining wards (wards one, two, and four), a total visitor ban was also introduced for these wards.

HCWs worked in set teams and wore gowns, surgical mouth masks, and gloves when they expected to get in close contact (within 1.5 m) with COVID-19 positive residents or their surroundings. Additionally, hand hygiene among HCWs was intensified. HCWs were regularly briefed with updates on a closed online portal, by team meetings with social distancing, and by newsletters shared via internal e-mail.

Since the implementation of these infection control measures, only one additional resident tested positive for COVID-19 on the 31st of March, after which no more residents have been infected, hospitalized, or have died due to complications from COVID-19 up to 8 weeks after the last infection. As a result, this LTCF was among the first 37 of 139 LTCFs in our region to allow visitors again.

### Cross-border policy discrepancies

Both international and national case definitions for suspect cases at the start of the COVID pandemic was based on a manifestation of disease like pneumonia rather than diseases with various manifestations ranging from mild to severe. Therefore, the case definition included a sudden onset of COVID symptoms including fever, as well as a history of travel or residence in a country or area reporting local or community transmission, or have had to be in close contact with a confirmed, or probable COVID-19 case [5]. This definition however, may not be applicable for our border region. At the moment of the LTCF outbreak, Heinsberg was not included as an area reporting local or community transmission. This means that, according to the national case definition at the time, suspect cases in the surrounding area of Heinsberg could be wrongfully dismissed as a potential COVID case due to a lack of relevant travel history. Therefore, in Limburg, we adhered to a regional case definition which differed from the national case definition.

First, we included travel to or from Gangelt (Heinsberg) as an area reporting local or community transmission. Second, a more sensitive case definition for COVID-19 suspected cases was used for the residents of the LTCF involved, which meant any respiratory symptoms or fever, including subfebrility (defined as temperature between 37.5 en 38 degrees Celsius) was seen as a suspect COVID case.

**Fig. 3:**
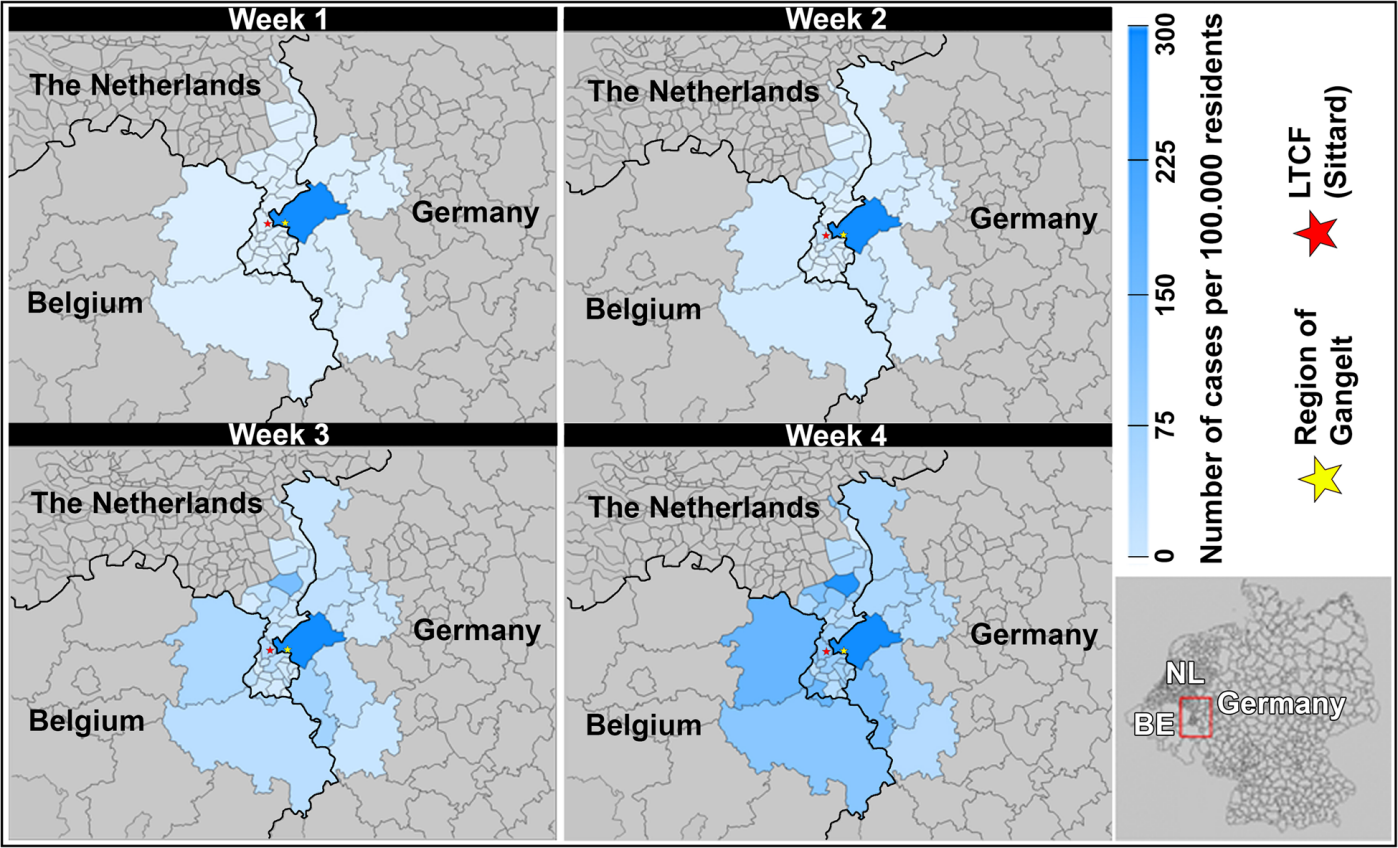
A map of the border region of Sittard and Gangelt showing the cumulative number of COVID-19 cases at four different time points in March 2020, the Netherlands. The different shades of blue represent the cumulative number of confirmed COVID cases per 100.000 residents for the first (days 01–07), second (days 01–14), third (days 01–21), and fourth (days 01–28) week of March. Greyed out regions were considered not relevant for this study. Therefore no colour has been assigned to them. The number of cases are based on the reported number of cases by the Rijksinstituut voor Volksgezondheid en Milieu (RIVM), Robert Koch Institut (RKI) and ScienSano for the Netherlands, Germany, and Belgium respectively. LTCF = long-term care facility. This figure is created by the first author and is owned by the public health service South Limburg

A series of geographical maps of the border region of Sittard and Gangelt is shown in Fig. 3, covering the first month of detected COVID-19 transmission. These four maps show the cumulative increase in the number of COVID-19 cases in our cross-border region at four different time points in March 2020.

## Discussion

Complete genome sequencing of the SARS-COV-2 samples of the eleven LTCF residents from Dutch Limburg showed complete clustering with virus strains found in the neighbouring German region of Heinsberg. This is in line with the open-border region of Limburg and Heinsberg characterized by cross-border commuter activity in both directions. Given the mutual carnival festivities on both sides of the border, the initial outbreak of COVID-19 in Heinsberg was accompanied by intensified cross-border movements. Though it has not been possible to identify the actual index case who introduced the SARS-CoV-2 virus into the LTCF, it seems plausible that the index was a (pre-)symptomatic carrier who picked up the virus through direct involvement in (carnival) activities across the border, or was a secondary contact, especially since pre-symptomatic cases and pre-symptomatic transmission have been reported [18, 19] and spreading of the virus while being asymptomatic cannot be excluded. Most probably, this carrier would have been a visitor to the LTCF, or a HCW.

Furthermore, this cross-border outbreak showed that (inter) national guidelines are not always sufficient or fully applicable in a border-region setting. Because Gangelt (Heinsberg) was not labelled as a COVID-19 endemic area during this outbreak, prior visits to this transmission area would not be categorized as a risk area, which could lead to many missed cases. As such, the (inter)national definition for suspect COVID cases at that moment in time turned out to be inappropriate for this specific region. In response to the cross-border situation in Gangelt, several guidelines were adjusted on a regional level by the public health service, as well as the regional hospital. For example, the physicians in the region were uneasy with the diagnostic testing for SARS-CoV-2 for admitted patients with pneumonia only when these cases were unexplained, as in most cases of community acquired pneumonia a causal agent often goes undetected. Therefore, even when the case definition for admitted cases was not completely met, a test for COVID-19 was immediately requested upon admission in the regional hospital. Nevertheless, deviation from national guidelines and international agreements was limited. Our results highlight the need for cross-border collaboration and adjustment of national policy to emerging region-specific needs along borders in order to establish coordinated implementation of infection control measures to limit the spread of COVID-19.

Many studies have examined the effect of travel restrictions such as closing the border or limiting air travel between countries [11, 12, 20–22]. However, literature on cross-border spread of COVID-19 is scarce. One of the few studies reporting on cross-border regions with regards to COVID, reported cross-border transmission despite a lockdown of the country and imposing border restrictions [13]. A British study which analysed the transmission of COVID in bordering districts in England during the first COVID wave showed similar results. The lockdown managed to reduce cross-district movement, but not entirely. Authors report that commute to work between districts was seen as a major cause for SARS-CoV-2 spread [14]. Although some similarities in findings exist, the authors did not describe a country cross-border region.

With regards to the LTCF, we observed a number of cases among LTCF residents in a cross-sectional analysis following two hospitalized residents who tested positive for COVID-19. Many of the residents who tested positive did not meet the clinical criteria for suspect cases of COVID-19 at that time [5]. Symptoms were reported only in about two third of the cases, and tended to be generally mild. Furthermore, CT values of asymptomatic (and possible pre-symptomatic) residents seem to be somewhat higher compared to symptomatic residents. This suggests that asymptomatic and pre-symptomatic residents could have accelerated further spread of the virus within the LTCF. However, due to the limited number of randomly tested residents, we cannot estimate the extent to which residents or HCWs could have attributed to further spread of the virus.

A study on COVID-19 within LTCFs by Arons et al. reports similar findings [23]. In a COVID-19 outbreak in a skilled nursing home in Washington, 48 out of 76 participating residents (63%) tested positive, of which 27 were asymptomatic at the time of testing, and 24 subsequently developed symptoms [23, 24]. A large cohort of symptomatic HCWs showed general non-respiratory symptoms including muscle ache, ocular pain, general malaise, headache, extreme tiredness, and fever were most frequently reported, as opposed to respiratory symptoms such as cough and sneezing [25]. Furthermore, nursing home residents seem to develop nonspecific symptoms prior to developing symptoms typical for COVID-19 [26, 27]. Reported nonspecific symptoms included, but are not limited to, a decrease in appetite, fatigue, confusion, and subfebrility or a low fever, which is often followed by respiratory symptoms [26]. Even after developing typical COVID-19 symptoms, residents may test negative due to respiratory or cardiovascular causes for their symptoms [27]. This illustrates the difficulty of screening for COVID-19 based on specific clinical signs. This is especially troublesome since identifying (a)symptomatic cases will help to minimise an outbreak and the overall impact of COVID-19 [28, 29]. Multiple studies conclude that infection-control strategies focused solely on symptomatic residents were not sufficient to prevent transmission after SARS-CoV-2 virus introduction, and urge for a recurring mass testing policy not limited to symptomatic residents, as well as regular check-ups to identify symptoms [23, 26, 28, 30–32].

This study extensively describes the first LTCF outbreak in the Netherlands from a cross-border perspective. By sequencing strains from the LTCF as well as the regional hospital, we were able to virtually prove cross-border transmission. Furthermore, this study describes the challenges a cross-border region encounters when dealing with a health crisis such as COVID-19. We were able to highlight the need for cross-border collaboration and adjustment of national policy to emerging needs along borders, which are applicable to other infectious diseases in a cross-border setting. However, we also encountered several limitations in the investigation of the outbreak. Due to a large difference in (mild) types of symptoms, it was difficult to pinpoint the onset of disease for positively tested residents, which hampered us in presenting a trustworthy epicurve. Instead, we have given a detailed description of events surrounding the LTCF. Additionally, although we were able to verify any new infections or deaths up to 8 weeks after the last detected infection, we were not able to acquire any follow-up data on symptoms, which made it impossible to categorize any asymptomatic residents as pre-symptomatic residents. In addition, a sample size of 99 residents is too small to draw any decisive conclusions, and the results presented in the manuscript should be interpreted as such. This is especially the case for the CT-values of the number of asymptomatic (and potentially pre-symptomatic) residents found (*n* = 3). Lastly, although it is very likely that the introduction of the SARS-CoV-2 virus was caused by an asymptomatic or pre-symptomatic HCW or visitor, the transmission route into the LTCF could not be identified with certainty. In order to give more support to the likelihood that cross-border transmission took place, we sequenced eleven samples from positive residents, as well as three positive patients from the regional hospital, which showed a strong similarity between sequences from Limburg and Heinsberg.

## Conclusions

In conclusion, the LTCF residents who tested positive did not meet the (inter)national criteria for suspect cases of COVID-19. Hence, adjusted criteria should be formed with regional- and cross-border partners to limit potential spread of the SARS-CoV-2 virus. Moreover, whole genome sequencing can help to identify and resolve potential transmission clusters. Given the clustering sequences found between the LTCF residents and the Heinsberg samples, introduction of the SARS-CoV-2 virus by a pre-symptomatic visitor or HCW with direct or indirect cross-border contacts, seems highly likely. The cross-border nature of this outbreak underlines the importance of sharing information with cross-border partners, as well as adjustment of national policy to emerging region-specific needs, in order to establish a coordinated implementation of infection control measures in the region to limit the spread of infectious diseases.

## Supplementary Information

**Additional file 1.** The complete phylogenetic tree (.pdf file). The full phylogenetic tree of COVID-19 cases at the time of testing (March 2020), including the sequenced samples from the LTCF.

## Data Availability

Nucleic acid sequence data has been shared with the Global Initiative on Sharing All Influenza Data (GISAID) database and can be accessed by registered GISAID users under the accession numbers EPI_ISL_415464, EPI_ISL_461001, EPI_ISL_461002, EPI_ISL_461003, EPI_ISL_461051, EPI_ISL_461052, EPI_ISL_461053, EPI_ISL_461054, EPI_ISL_461055, EPI_ISL_461056, EPI_ISL_461057, EPI_ISL_461058, EPI_ISL_461059, and EPI_ISL_461060. The datasets generated and/or analysed during the current study are available in the Synapse repository, https://www.synapse.org/#!Synapse:syn23532550/, under the Synapse ID ‘syn23532550’.

## Abbreviations

COVID-19: Coronavirus disease 2019
LTCF: Long-term care facility
HCWs: Health care workers
RT-PCR: Real-time Polymerase Chain Reaction
RdRp: RNA-dependent RNA polymerase
CMV: Murine cytomegalovirus
CT value: Cycle Threshold value
GISAID: Global Initiative on Sharing All Influenza Data
