# Supplementary material for: COVID-19: first long-term care facility outbreak in the Netherlands following cross-border introduction from Germany, March 2020
Source: BMC Infect Dis. 2021 May 4;21:418. doi: 10.1186/s12879-021-06093-9 (PMC8094983; doi:10.1186/s12879-021-06093-9)

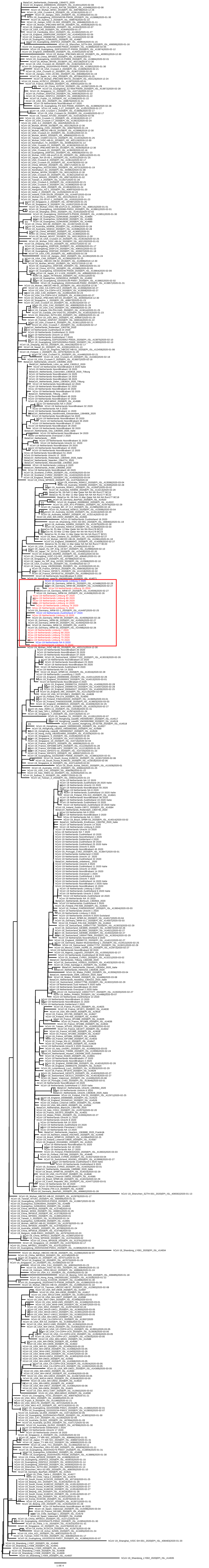

Supplement: Supplementary file 1 — Additional file 1. The complete phylogenetic tree (.pdf file). The full phylogenetic tree of COVID-19 cases at the time of testing (March 2020), including the sequenced samples from the LTCF. [file 12879_2021_6093_MOESM1_ESM.pdf]
